# *rac*-1,2,3,4-Tetra­hydro-1,4-methano­anthracene-6,7-dicarbonitrile

**DOI:** 10.1107/S1600536811047611

**Published:** 2011-11-16

**Authors:** Kew-Yu Chen, Ming-Jen Chang, Tzu-Chien Fang, Ming-Hui Luo, Hsing-Yang Tsai

**Affiliations:** a Department of Chemical Engineering, Feng Chia University, 40724 Taichung, Taiwan

## Abstract

The title compound, C_17_H_12_N_2_, comprises a norbornane unit having a dicyanona­phthalene ring fused on one side. Both cyano groups are twisted slightly out of the plane of the naphthalene ring system [C—C—C—C torsion angle = 1.9 (2)°]. In the crystal, inversion-related mol­ecules are linked by pairs of weak C—H⋯N hydrogen bonds, forming dimers.

## Related literature

For the spectroscopy of the title compound and its prepartion, see: Chen *et al.* (2006 ▶). For the spectroscopy and electronic device applications of rigid oligo-norbornyl compounds, see: Chen *et al.* (2002 ▶); Chow *et al.* (2005 ▶); Foitzik *et al.* (2009 ▶); Jansen *et al.* (2010 ▶); Tang *et al.* (2009 ▶). For related structures, see: Çelik *et al.* (2006 ▶); Chen *et al.* (2011 ▶); Lough *et al.* (2006 ▶). For puckering parameters, see: Cremer & Pople (1975 ▶). For graph-set theory, see: Bernstein *et al.* (1995 ▶).
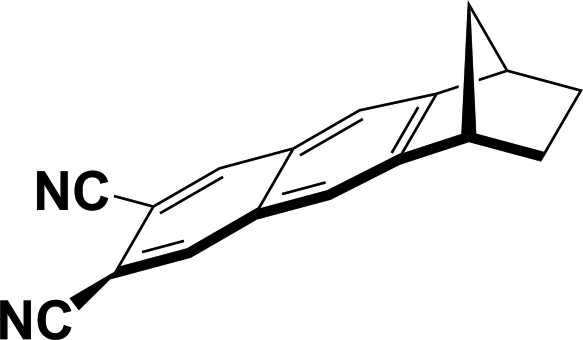


## Experimental

### 

#### Crystal data

C_17_H_12_N_2_
*M* *_r_* = 244.29
Triclinic, 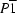

*a* = 6.1019 (4) Å
*b* = 10.7078 (6) Å
*c* = 11.3928 (7) Å
α = 65.173 (5)°
β = 84.768 (5)°
γ = 73.900 (5)°
*V* = 648.82 (7) Å^3^
*Z* = 2
Mo *K*α radiation
μ = 0.08 mm^−1^
*T* = 297 K
0.64 × 0.52 × 0.48 mm

#### Data collection

Bruker SMART 1000 CCD detector diffractometer
5692 measured reflections
2997 independent reflections
1707 reflections with *I* > 2σ(*I*)
*R*_int_ = 0.020

#### Refinement

*R*[*F* ^2^ > 2σ(*F* ^2^)] = 0.041
*wR*(*F* ^2^) = 0.102
*S* = 1.00
2997 reflections
172 parameters
1 restraint
H-atom parameters constrained
Δρ_max_ = 0.14 e Å^−3^
Δρ_min_ = −0.12 e Å^−3^

### 

Data collection: *SMART* (Bruker, 2007 ▶); cell refinement: *SAINT* (Bruker, 2007 ▶); data reduction: *SAINT*; program(s) used to solve structure: *SHELXS97* (Sheldrick, 2008 ▶); program(s) used to refine structure: *SHELXL97* (Sheldrick, 2008 ▶); molecular graphics: *ORTEP-3 for Windows* (Farrugia, 1997 ▶); software used to prepare material for publication: *WinGX* (Farrugia, 1999 ▶).

## Supplementary Material

Crystal structure: contains datablock(s) I, global. DOI: 10.1107/S1600536811047611/xu5368sup1.cif

Structure factors: contains datablock(s) I. DOI: 10.1107/S1600536811047611/xu5368Isup2.hkl

Additional supplementary materials:  crystallographic information; 3D view; checkCIF report

## Figures and Tables

**Table 1:** Hydrogen-bond geometry (Å, °)

*D*—H⋯*A*	*D*—H	H⋯*A*	*D*⋯*A*	*D*—H⋯*A*
C4—H4*A*⋯N1^i^	0.93	2.61	3.505 (2)	162

Symmetry code: (i) 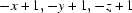.

**Fig. 1.**
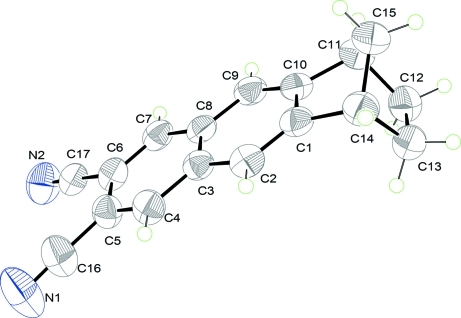
The molecular structure of the title compound, showing 50% probability displacement ellipsoids.

**Fig. 2.**
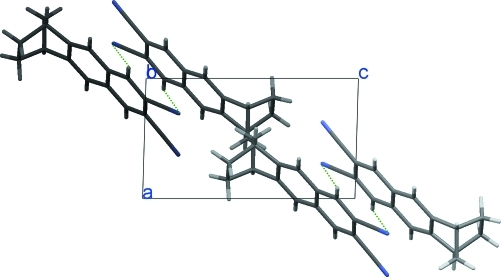
A section of the crystal packing of the title compound, viewed down the b axis. Green dashed lines denote the intermolecular C—H···N hydrogen bonds.

## supplementary crystallographic information

### Comment

Donor-acceptor chromophores linked by the rigid norbornane have been synthesized (Foitzik *et al.*, 2009; Jansen *et al.*, 2010; Tang *et al.*, 2009). The highly symmetrical structures reduce the complexity due to the constraint of geometrical and conformational variations. The rates of photoinduced electron transfer reactions across linearly fused oligo-norbornyl spacer groups have been estimated (Chen *et al.*, 2002; Chow *et al.*, 2005). The ET rates were found to correlate well with both D—A distance and solvent polarities. Atoms C11 and C14 of the title compound are chiral centers, but their relative configurations are opposite. The racemate was prepared as a model compound for investigations of the intramolecular electron transfer reactions (Chen *et al.*, 2006).

The structure of the title compound comprises a norbornane unit having a dicyanonaphthalene ring fused on one side (Figure 1). The naphthalene is essentially planar with a maximum deviation of 0.039 (2)° for atom C5. Whereas both cyano groups are slightly twisted out of the plane of the naphthalene ring (1.9 (2)° of C17–C6–C5–C16). The puckering parameters (Cremer & Pople, 1975) of the five-membered rings *A* (C1/C10/C11/C15/C14) and *B* (C11–C15) are *Q*_2_ = 0.5621 (17)Å and φ_2_ = 287.97 (16)°, and *Q*_2_ = 0.6013 (17)Å and φ_2_ = 144.60 (16)°, respectively. These results are slightly different from those of previous studies on other norbornane derivatives (Çelik, *et al.*, 2006; Chen, *et al.*, 2011; Lough, *et al.*, 2006). In the crystal structure (Figure 2), inversion-related molecules are linked by a pair of C—H···N hydrogen bonds (Table 1), forming a cyclic dimers with *R*_2_^2^(10) graph-set motif (Bernstein *et al.*, 1995). The C—H···π interactions are also observed (2.81 Å of C13—H13B···*Cg*3 distance, symmetry code: -*x*, -*y*, -*z* + 1, where *Cg*3 is the centroid of the C1–C3/C8–C10 ring), and further stabilize the crystal structure.

### Experimental

The title compound was synthesized according to the literature (Chen *et al.*, 2006). Colorless needle-shaped crystals suitable for the crystallographic studies reported here were isolated over a period of six weeks by slow evaporation from the chloroform solution.

### Refinement

The C bound H atoms positioned geometrically (C—H = 0.93–0.98 Å) and allowed to ride on their parent atoms, with *U*_iso_(H) = 1.2*U*_eq_(C).

### Crystal data

**Table:**

C_17_H_12_N_2_	*Z* = 2
*M**_r_* = 244.29	*F*(000) = 256
Triclinic, *P*1	*D*_x_ = 1.250 Mg m^−^^3^
Hall symbol: -P 1	Mo *K*α radiation, λ = 0.71073 Å
*a* = 6.1019 (4) Å	Cell parameters from 2453 reflections
*b* = 10.7078 (6) Å	θ = 3.5–29.1°
*c* = 11.3928 (7) Å	µ = 0.08 mm^−^^1^
α = 65.173 (5)°	*T* = 297 K
β = 84.768 (5)°	Parallelepiped, colorless
γ = 73.900 (5)°	0.64 × 0.52 × 0.48 mm
*V* = 648.82 (7) Å^3^	

### Data collection

**Table:**

Bruker SMART 1000 CCD detector diffractometer	1707 reflections with *I* > 2σ(*I*)
Radiation source: fine-focus sealed tube	*R*_int_ = 0.020
graphite	θ_max_ = 29.2°, θ_min_ = 3.5°
ω scans	*h* = −8→8
5692 measured reflections	*k* = −14→14
2997 independent reflections	*l* = −14→15

### Refinement

**Table:**

Refinement on *F*^2^	Primary atom site location: structure-invariant direct methods
Least-squares matrix: full	Secondary atom site location: difference Fourier map
*R*[*F*^2^ > 2σ(*F*^2^)] = 0.041	Hydrogen site location: inferred from neighbouring sites
*wR*(*F*^2^) = 0.102	H-atom parameters constrained
*S* = 1.00	*w* = 1/[σ^2^(*F*_o_^2^) + (0.050*P*)^2^] where *P* = (*F*_o_^2^ + 2*F*_c_^2^)/3
2997 reflections	(Δ/σ)_max_ < 0.001
172 parameters	Δρ_max_ = 0.14 e Å^−^^3^
1 restraint	Δρ_min_ = −0.12 e Å^−^^3^

### Special details

**Table:**

Geometry. All e.s.d.'s (except the e.s.d. in the dihedral angle between two l.s. planes) are estimated using the full covariance matrix. The cell e.s.d.'s are taken into account individually in the estimation of e.s.d.'s in distances, angles and torsion angles; correlations between e.s.d.'s in cell parameters are only used when they are defined by crystal symmetry. An approximate (isotropic) treatment of cell e.s.d.'s is used for estimating e.s.d.'s involving l.s. planes.
Refinement. Refinement of *F*^2^ against ALL reflections. The weighted *R*-factor *wR* and goodness of fit *S* are based on *F*^2^, conventional *R*-factors *R* are based on *F*, with *F* set to zero for negative *F*^2^. The threshold expression of *F*^2^ > σ(*F*^2^) is used only for calculating *R*-factors(gt) *etc*. and is not relevant to the choice of reflections for refinement. *R*-factors based on *F*^2^ are statistically about twice as large as those based on *F*, and *R*- factors based on ALL data will be even larger.

### Fractional atomic coordinates and isotropic or equivalent isotropic displacement parameters (Å^2^)

**Table:**

	*x*	*y*	*z*	*U*_iso_*/*U*_eq_	
N1	0.2097 (3)	0.55552 (16)	0.34283 (14)	0.1097 (6)	
N2	−0.1343 (2)	0.29042 (13)	0.33818 (12)	0.0842 (4)	
C1	0.78769 (19)	−0.08604 (13)	0.86297 (11)	0.0499 (3)	
C2	0.74313 (19)	0.04981 (12)	0.77112 (11)	0.0502 (3)	
H2A	0.8289	0.1101	0.7692	0.060*	
C3	0.56549 (19)	0.09894 (12)	0.67840 (11)	0.0455 (3)	
C4	0.5038 (2)	0.24135 (12)	0.58626 (11)	0.0538 (3)	
H4A	0.5862	0.3034	0.5841	0.065*	
C5	0.3264 (2)	0.29073 (13)	0.50013 (11)	0.0534 (3)	
C6	0.2009 (2)	0.19691 (13)	0.50028 (11)	0.0502 (3)	
C7	0.2591 (2)	0.05771 (13)	0.58871 (11)	0.0516 (3)	
H7A	0.1780	−0.0038	0.5879	0.062*	
C8	0.43752 (19)	0.00511 (12)	0.68053 (10)	0.0459 (3)	
C9	0.4902 (2)	−0.13666 (12)	0.77692 (11)	0.0537 (3)	
H9A	0.4095	−0.1996	0.7790	0.064*	
C10	0.6589 (2)	−0.17993 (12)	0.86607 (11)	0.0521 (3)	
C11	0.7439 (2)	−0.31536 (13)	0.98405 (12)	0.0652 (4)	
H11A	0.7136	−0.4008	0.9845	0.078*	
C12	0.6585 (2)	−0.28095 (14)	1.10178 (12)	0.0680 (4)	
H12A	0.4960	−0.2353	1.0934	0.082*	
H12B	0.6900	−0.3666	1.1818	0.082*	
C13	0.7968 (2)	−0.17846 (14)	1.09723 (12)	0.0628 (4)	
H13A	0.8894	−0.2172	1.1757	0.075*	
H13B	0.6968	−0.0861	1.0861	0.075*	
C14	0.9474 (2)	−0.16675 (13)	0.97878 (12)	0.0596 (3)	
H14A	1.0812	−0.1317	0.9752	0.071*	
C15	0.9970 (3)	−0.31873 (14)	0.98683 (14)	0.0739 (4)	
H15A	1.0779	−0.3294	0.9128	0.089*	
H15B	1.0768	−0.3906	1.0666	0.089*	
C16	0.2613 (2)	0.43837 (17)	0.41097 (14)	0.0729 (4)	
C17	0.0154 (2)	0.24891 (14)	0.40941 (13)	0.0598 (3)	

### Atomic displacement parameters (Å^2^)

**Table:**

	*U*^11^	*U*^22^	*U*^33^	*U*^12^	*U*^13^	*U*^23^
N1	0.1226 (13)	0.0715 (9)	0.1080 (11)	−0.0383 (8)	−0.0460 (10)	0.0062 (8)
N2	0.0804 (9)	0.1061 (10)	0.0719 (8)	−0.0326 (8)	−0.0084 (7)	−0.0352 (8)
C1	0.0473 (7)	0.0524 (7)	0.0519 (7)	−0.0076 (6)	0.0070 (6)	−0.0279 (6)
C2	0.0455 (7)	0.0567 (8)	0.0545 (7)	−0.0178 (6)	0.0066 (6)	−0.0271 (6)
C3	0.0461 (7)	0.0493 (7)	0.0457 (7)	−0.0166 (5)	0.0097 (5)	−0.0232 (6)
C4	0.0584 (8)	0.0549 (8)	0.0534 (7)	−0.0280 (6)	0.0069 (6)	−0.0202 (6)
C5	0.0573 (8)	0.0543 (8)	0.0482 (7)	−0.0205 (6)	0.0036 (6)	−0.0174 (6)
C6	0.0545 (7)	0.0589 (8)	0.0437 (7)	−0.0201 (6)	0.0072 (5)	−0.0250 (6)
C7	0.0593 (8)	0.0613 (8)	0.0492 (7)	−0.0277 (6)	0.0091 (6)	−0.0313 (7)
C8	0.0522 (7)	0.0497 (7)	0.0446 (7)	−0.0177 (5)	0.0104 (6)	−0.0270 (6)
C9	0.0672 (9)	0.0491 (7)	0.0563 (8)	−0.0232 (6)	0.0096 (7)	−0.0292 (6)
C10	0.0610 (8)	0.0454 (7)	0.0526 (7)	−0.0091 (6)	0.0064 (6)	−0.0267 (6)
C11	0.0831 (11)	0.0438 (7)	0.0639 (9)	−0.0080 (6)	−0.0018 (7)	−0.0222 (6)
C12	0.0800 (10)	0.0589 (8)	0.0550 (8)	−0.0135 (7)	0.0037 (7)	−0.0173 (7)
C13	0.0669 (9)	0.0625 (8)	0.0546 (8)	−0.0064 (7)	−0.0032 (6)	−0.0256 (7)
C14	0.0512 (8)	0.0602 (8)	0.0626 (8)	−0.0024 (6)	−0.0016 (6)	−0.0280 (7)
C15	0.0777 (11)	0.0603 (9)	0.0685 (9)	0.0089 (7)	−0.0018 (7)	−0.0284 (7)
C16	0.0760 (11)	0.0660 (10)	0.0707 (10)	−0.0296 (8)	−0.0158 (8)	−0.0122 (8)
C17	0.0626 (8)	0.0724 (9)	0.0521 (8)	−0.0266 (7)	0.0038 (6)	−0.0279 (7)

### Geometric parameters (Å, °)

**Table:**

N1—C16	1.1327 (16)	C8—C9	1.4169 (15)
N2—C17	1.1390 (14)	C9—C10	1.3557 (15)
C1—C2	1.3562 (15)	C9—H9A	0.9300
C1—C10	1.4256 (16)	C10—C11	1.5006 (16)
C1—C14	1.4990 (16)	C11—C15	1.5380 (19)
C2—C3	1.4119 (15)	C11—C12	1.5450 (17)
C2—H2A	0.9300	C11—H11A	0.9800
C3—C4	1.4065 (15)	C12—C13	1.5414 (19)
C3—C8	1.4252 (15)	C12—H12A	0.9700
C4—C5	1.3616 (16)	C12—H12B	0.9700
C4—H4A	0.9300	C13—C14	1.5419 (18)
C5—C6	1.4213 (16)	C13—H13A	0.9700
C5—C16	1.4373 (19)	C13—H13B	0.9700
C6—C7	1.3687 (16)	C14—C15	1.5346 (18)
C6—C17	1.4296 (18)	C14—H14A	0.9800
C7—C8	1.4035 (15)	C15—H15A	0.9700
C7—H7A	0.9300	C15—H15B	0.9700
			
C2—C1—C10	120.79 (10)	C10—C11—C12	106.33 (9)
C2—C1—C14	132.93 (11)	C15—C11—C12	100.68 (11)
C10—C1—C14	106.15 (11)	C10—C11—H11A	115.7
C1—C2—C3	119.46 (11)	C15—C11—H11A	115.7
C1—C2—H2A	120.3	C12—C11—H11A	115.7
C3—C2—H2A	120.3	C13—C12—C11	103.09 (11)
C2—C3—C4	121.51 (10)	C13—C12—H12A	111.1
C2—C3—C8	119.94 (11)	C11—C12—H12A	111.1
C4—C3—C8	118.49 (10)	C13—C12—H12B	111.1
C5—C4—C3	121.73 (11)	C11—C12—H12B	111.1
C5—C4—H4A	119.1	H12A—C12—H12B	109.1
C3—C4—H4A	119.1	C12—C13—C14	103.60 (10)
C4—C5—C6	119.99 (11)	C12—C13—H13A	111.0
C4—C5—C16	120.35 (11)	C14—C13—H13A	111.0
C6—C5—C16	119.63 (11)	C12—C13—H13B	111.0
C7—C6—C17	120.93 (11)	C14—C13—H13B	111.0
C7—C6—C5	119.19 (10)	H13A—C13—H13B	109.0
C17—C6—C5	119.87 (11)	C1—C14—C13	105.82 (10)
C6—C7—C8	121.89 (10)	C1—C14—C15	100.52 (10)
C6—C7—H7A	119.1	C13—C14—C15	100.81 (11)
C8—C7—H7A	119.1	C1—C14—H14A	115.8
C7—C8—C9	122.15 (10)	C13—C14—H14A	115.8
C7—C8—C3	118.67 (10)	C15—C14—H14A	115.8
C9—C8—C3	119.17 (10)	C11—C15—C14	94.37 (10)
C10—C9—C8	119.52 (11)	C11—C15—H15A	112.9
C10—C9—H9A	120.2	C14—C15—H15A	112.9
C8—C9—H9A	120.2	C11—C15—H15B	112.9
C9—C10—C1	121.11 (11)	C14—C15—H15B	112.9
C9—C10—C11	132.89 (12)	H15A—C15—H15B	110.3
C1—C10—C11	105.90 (10)	N1—C16—C5	178.55 (15)
C10—C11—C15	100.49 (11)	N2—C17—C6	179.15 (14)
			
C10—C1—C2—C3	0.63 (16)	C8—C9—C10—C11	174.59 (11)
C14—C1—C2—C3	−174.57 (11)	C2—C1—C10—C9	0.59 (17)
C1—C2—C3—C4	176.05 (10)	C14—C1—C10—C9	176.93 (10)
C1—C2—C3—C8	−1.11 (16)	C2—C1—C10—C11	−176.27 (10)
C2—C3—C4—C5	−177.21 (11)	C14—C1—C10—C11	0.07 (12)
C8—C3—C4—C5	0.00 (16)	C9—C10—C11—C15	149.70 (13)
C3—C4—C5—C6	−1.17 (18)	C1—C10—C11—C15	−33.97 (12)
C3—C4—C5—C16	177.24 (11)	C9—C10—C11—C12	−105.79 (15)
C4—C5—C6—C7	0.78 (17)	C1—C10—C11—C12	70.54 (13)
C16—C5—C6—C7	−177.64 (11)	C10—C11—C12—C13	−68.26 (13)
C4—C5—C6—C17	−179.75 (11)	C15—C11—C12—C13	36.12 (12)
C16—C5—C6—C17	1.83 (18)	C11—C12—C13—C14	−0.59 (12)
C17—C6—C7—C8	−178.66 (11)	C2—C1—C14—C13	105.11 (14)
C5—C6—C7—C8	0.80 (17)	C10—C1—C14—C13	−70.59 (11)
C6—C7—C8—C9	176.37 (10)	C2—C1—C14—C15	−150.36 (13)
C6—C7—C8—C3	−1.95 (16)	C10—C1—C14—C15	33.94 (13)
C2—C3—C8—C7	178.78 (10)	C12—C13—C14—C1	69.09 (12)
C4—C3—C8—C7	1.53 (15)	C12—C13—C14—C15	−35.23 (12)
C2—C3—C8—C9	0.42 (15)	C10—C11—C15—C14	52.20 (11)
C4—C3—C8—C9	−176.84 (10)	C12—C11—C15—C14	−56.81 (11)
C7—C8—C9—C10	−177.53 (11)	C1—C14—C15—C11	−52.13 (12)
C3—C8—C9—C10	0.78 (16)	C13—C14—C15—C11	56.39 (11)
C8—C9—C10—C1	−1.29 (17)		

### Hydrogen-bond geometry (Å, °)

**Table:**

*D*—H···*A*	*D*—H	H···*A*	*D*···*A*	*D*—H···*A*
C4—H4A···N1^i^	0.93	2.61	3.505 (2)	162

Symmetry codes: (i) −*x*+1, −*y*+1, −*z*+1.
